# A symptomatic cyamella in the popliteus tendon causing snapping knee: a case report and literature review

**DOI:** 10.1186/s12891-019-2882-8

**Published:** 2019-10-27

**Authors:** Shouwen Su, Yunxiang Lu, Yuxian Chen, Zhiyong Li

**Affiliations:** Department of Orthopedics, The Third Affiliated Hospital, Sun Yat-sen University, Guangzhou, Guangdong China

**Keywords:** Cyamella, Popliteus, Sesamoid, Snapping knee, Surgery, Knee joint

## Abstract

**Background:**

Cyamella,the sesamoid bones of the popliteus muscle, are rare in humans. Snapping knee is an uncommon problem which can be difficult to diagnose.

**Case presentation:**

In this case, we report a 24-year-old male with snapping knee caused by symptomatic cyamella in the popliteus tendon. A large cyamella was identified upon surgery and was removed. Postoperatively, the patient had immediate relief of preoperative symptoms, and there were no signs of recurrence after 1 years of follow-up.

**Conclusions:**

Although not previously suggested, symptomatic cyamella in the popliteus tendon should be considered as part of the differential diagnosis of the snapping knee.

## Background

Cyamellae, the sesamoid bones of the popliteus muscle, are rare in humans [1]. Snapping knee, which is defined as a patient hearing or feeling a snapping or popping of joints at some specific activity [2], is an uncommon problem and can be difficult to diagnose [3]. Some differentials for the snapping knee include the presence of a discoid meniscus [4], rheumatoid nodules [5], iliotibial band (ITB) friction [6], biceps femoris tendon [3, 7], semitendinosus and gracilis tendons [8], popliteus tendon [9, 10] and intra-articular pathological changes. Firstly in this case, we report a 24-year-old male with snapping knee caused by symptomatic cyamella in the popliteus tendon. Secondly, diagnosis and treatment of this rare pathology are discussed. The literature associated with symptomatic cyamella in the popliteus tendon are also reviewed.

## Case presentation

A 24-year-old male patient was presented to the outpatient department with a history of right lateral knee snapping and recurrent sensation of discomfort for the past 2 years. Snapping was elicited upon extending the knee and could be reproduced by applyng direct pressure on the posterolateral knee. There was no actual or previously sustained trauma noted on this patient.

The patient was initially and preliminarily diagnosed with knee joint plica syndrome and underwent arthroscopic surgery in a previous institution. This however did not relieve his symptoms. As the snapping continued, the patient became unable to tolerate physical activities or prolonged walking.

Upon thorough physical examination, full active range of motion was observed to be intact, but a reproducible audible and palpable snapping of the lateral knee when moving from flexion to extension. However, this was not consistently reproducible with passive range of motion. (see Additional file 1) Other special tests were negative except for the Cabot sign, which clearly produced a snapping sensation.

**Fig. 1:**
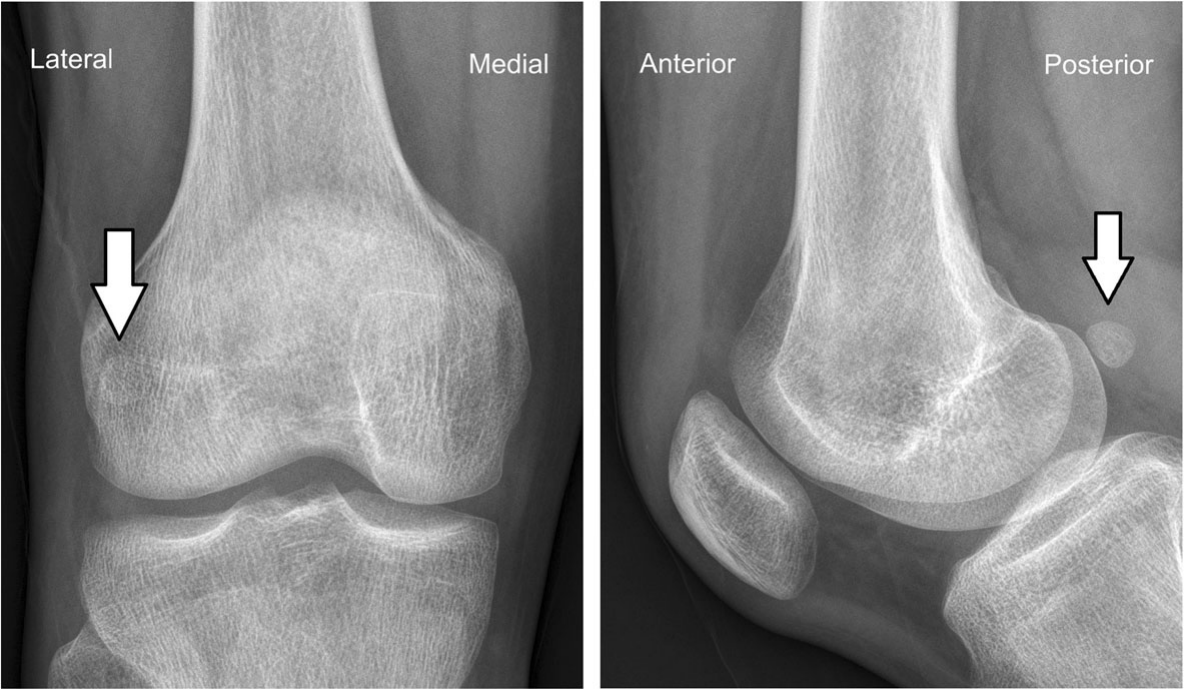
Radiograph of the right knee. The cyamella may be seen as a round osseous body (arrow). "Lateral" indicates lateral side of the knee, "Medial" indicates medial side of the knee

**Fig. 2:**
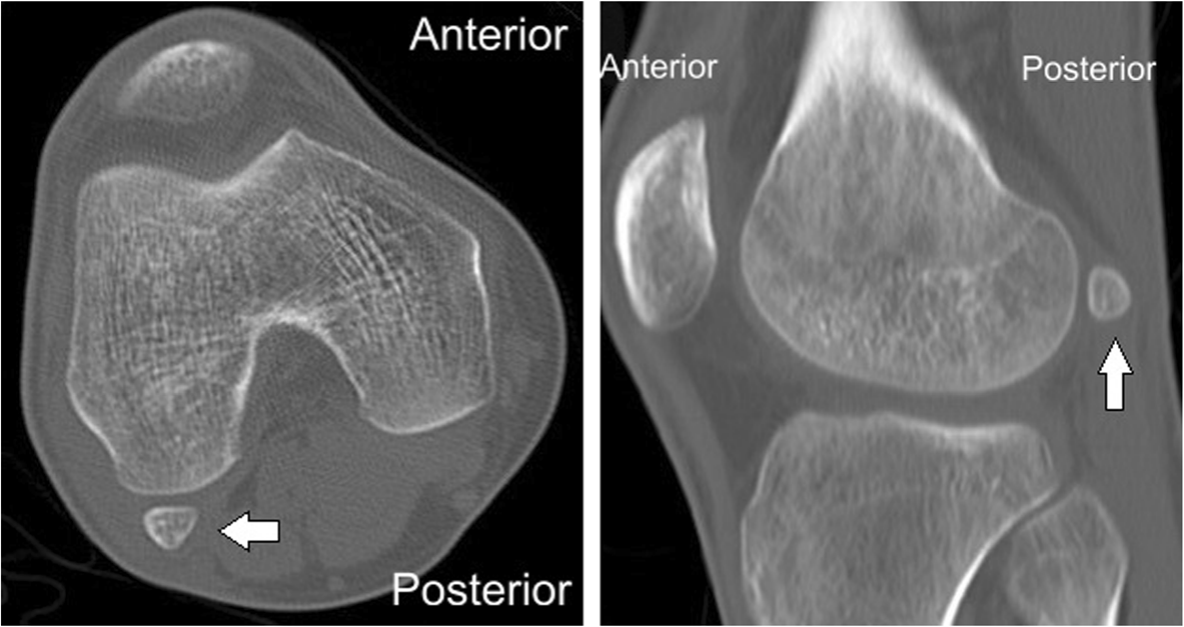
CT of the right knee. "Lateral" indicates lateral side of the knee, "Medial" indicates medial side of the knee, "Anterior" indicates anterior side of the knee, "Posterior" indicates posterior side of the knee. Arrow indicates the cyamella

**Fig. 3:**
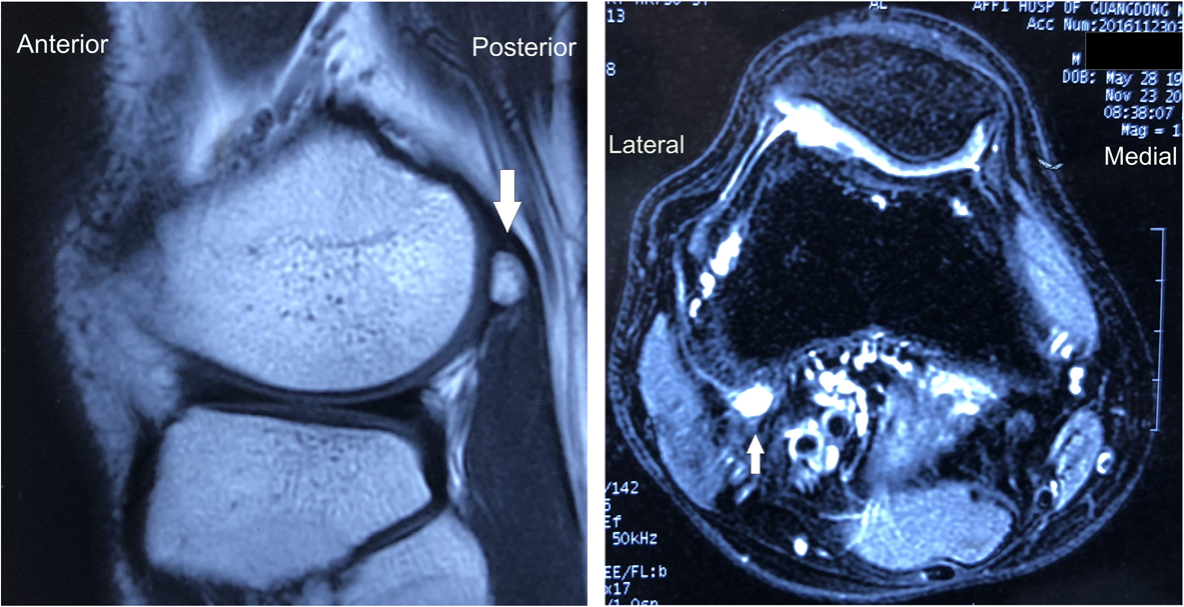
MRI scan of the right knee. The cyamella is shown with cortical and cancellous parts in the popliteal tendon posteriorsuperior. "Lateral" indicates lateral side of the knee, "Medial" indicates medial side of the knee, "Anterior" indicates anterior side of the knee, "Posterior" indicates posterior side of the knee. Arrow indicates the cyamella

X-rays presented a round osseous structure in the posterolateral part of the joint, similar to the normal sesamoid (Fig. 1). CT scanning and MRI were additionally conducted. No pathological results were obtained apart from a clearly visible ovoid-shaped bone that was located posterior and superior to the proximal musculo-tendinous intersection of the popliteus muscle. The sesamoid bone articulates non-cartilagenously with the lateral dorsal femoral condyle (Figs. 2 and 3). The popliteus muscle and tendon presented signs of inflammation. A steroid injection was administered into the snapping point but did not relieve his symptoms.

**Fig. 4:**
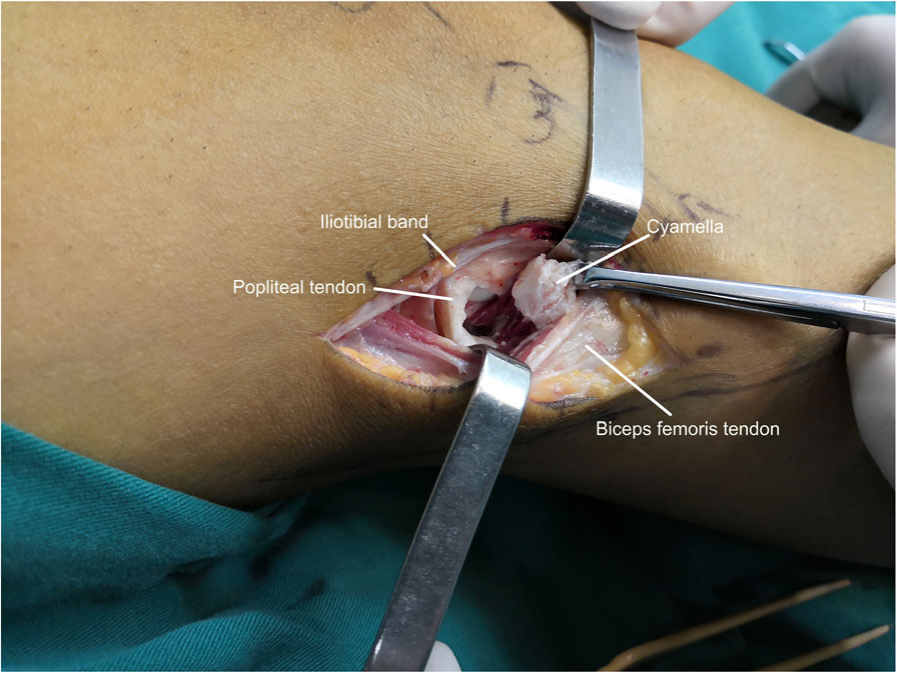
Excising the cyamella through a lateral incision, note the cyamella located near the musculo-tendinous intersection of the popliteus muscle

**Fig. 5:**
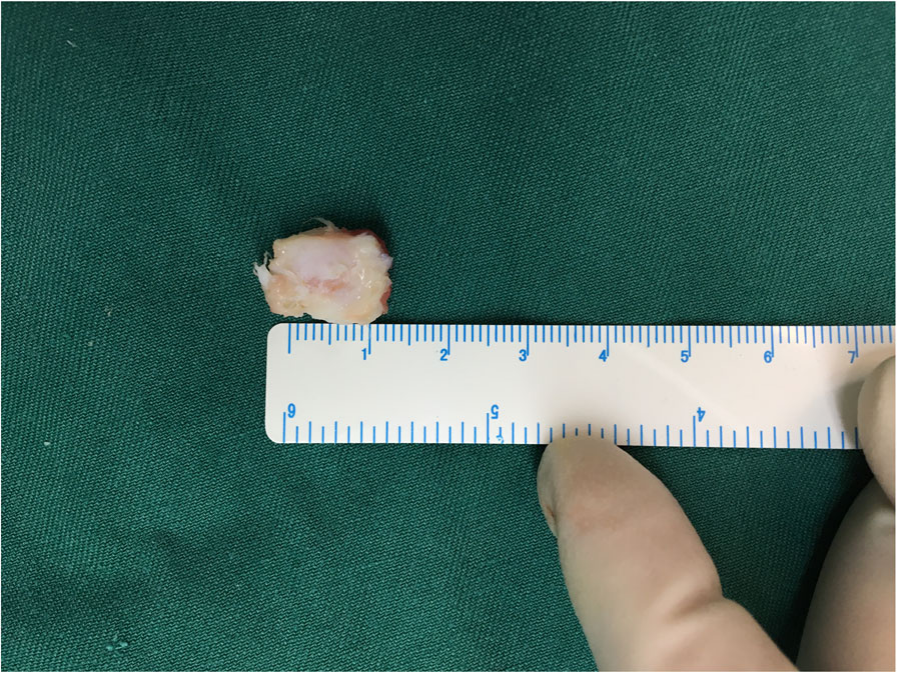
Excised cyamella

**Fig. 6:**
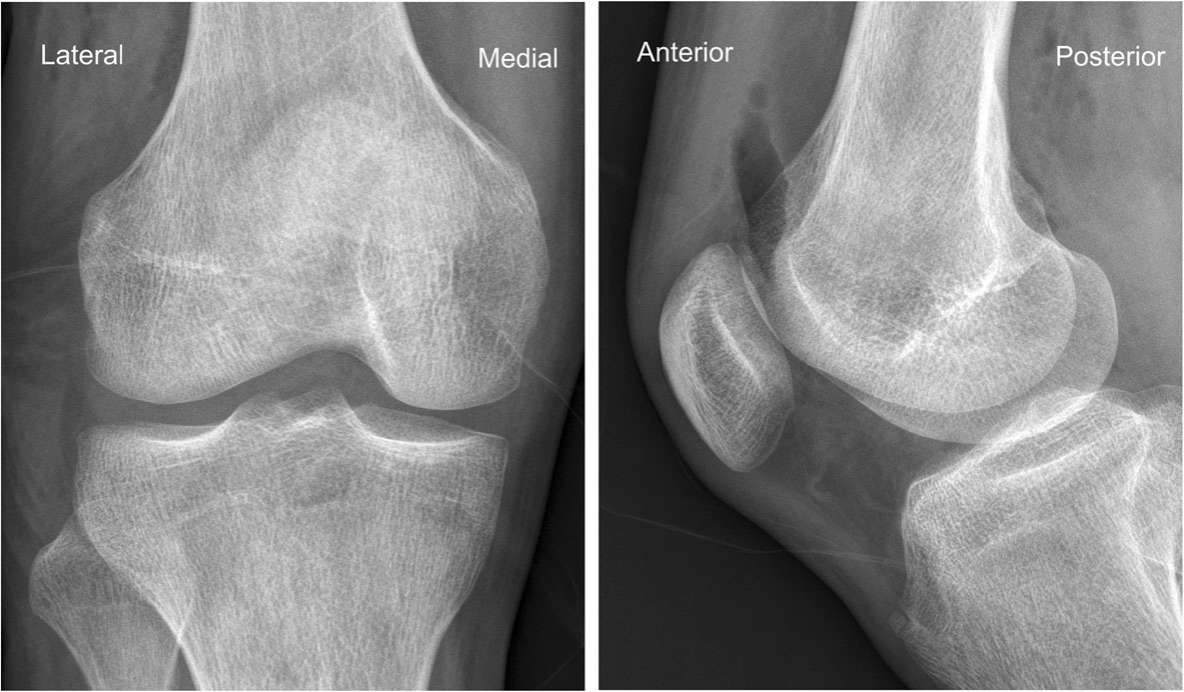
Postoperative radiograph after excision of cyamella. "Lateral" indicates lateral side of the knee, "Medial" indicates medial side of the knee, "Anterior" indicates anterior side of the knee, "Posterior" indicates posterior side of the knee

A posterior approach was used to incise the bone which lead us to discover that the biceps femoris tendon presented no pathological changes. Deeper in the popliteus tendon, a large cyamella was found. With the knee in a passive range of motion, we found there was snapping of the popliteus tendon over the cyamella. (see Additional file 2) An incision was made directly over the located area and the sesamoid bone was excised (see Additional file 3). The cyamella located near the musculo-tendinous intersection of the popliteus muscle (Fig. 4). The specimen measured 15*7*9 mm (Fig. 5). Radiographs following the procedure demonstrated removal of the cyamella (Fig. 6).

Postoperatively, the patient recovered well and had immediate relief after treatment. He was able to return to physical fitness activities at 8-week follow-up.

## Discussion and conclusions

Snapping knee is defined as a patient hearing or feeling a snapping or popping of joints at some specific activity [3]. Some differentials for the snapping knee include the presence of a discoid meniscus [4], rheumatoid nodules [5], iliotibial band (ITB) friction [6], biceps femoris tendon [7], semitendinosus and gracilis tendons [8], popliteus tendon [9, 10] and intra-articular pathological changes. Snapping of the knee caused by symptomatic cyamella in the popliteus tendon however, is extremely rare and has never been reported in the literature.

Cyamella, the sesamoid bones of the popliteus muscle, are rare in humans. While in animal research, they are thought to assist in muscle function by modifying pressure, diminishing friction and altering the direction of the pull [1]. Studies have shown a close interaction between intrinsic genetic factors and extrinsic epigenetic stimuli ultimately controls the development and evolution of sesamoid bones [11]. The cyamella is located in either the popliteus muscle or adjacent to its myotendinous junction. Other knee sesamoid bones include the patella and fabella. The fabella is located within the lateral head of the gastrocnemius muscle and posterior to the lateral femoral condyle [12].

In the present case, we found Cabot sign could reproduce the snapping while other spacial tests were unremarkable. The Cabot test is performed on the patient in a supine position with the involved knee flexed with the lower leg crossed over the contralateral leg. The patient is then asked to extend the knee while the examiner palpates the lateral joint line. The sign is considered positive when snapping is elicited upon knee extension. We noted that this is a typical physical examination for differential diagnosis of the snapping knee [10].

Isolated case reports include the imaging appearance of cyamella. CT showed cyamella to be a well-corticated ossicle with a hypodense center due to the presence of marrow fat [13]. On T1-, T2-, and T2*-weighted MRI scans, a cyamella presents as an ossicle with low signal intensity along its borders [14]. According to these results, we were able to exclude an osteochondral flake, a loose body or a periosseous calcification in this case, the diagnosis of a cyamella was given. Ultrasound has its unique value in diagnosis and evaluation of snapping knee. The benefit of musculoskeletal ultrasound includes quick examination, high accuracy and no radiation [15–17]. As the patient did MRI scans in the first place, we did not employ ultrasound for diagnosis.

The review of the literature available via the PubMed online data revealed four case reports associated with symptomatic cyamella in the popliteus tendon [18–21]. The results are summarized in Table 1.

**Table 1:** Details derived from four case reports

	Mishra.1996 [15]	Benthien.2010 [16]	Dheer.2012 [17]	Rehmatullah.2014 [18]
Age of patient	28Y	25Y	14Y	64Y
Symptoms	Unable to bear weight,swelling	Posterolateral knee pain,swelling and discomfort for 6 weeks	Lateral knee pain,swelling, inability to fully extend his knee	Intermittent knee pain for 4 months
History of trauma	Y	N	N	Y
Treatment	Arthroscopy	Physical therapy	Not mentioned	Hinge brace
Follow up	Asymptomatic after 6 weeks	Asymptomatic after 1 year	Not mentioned	Symptoms reoccurred once the brace was removed

The novelty of this article includes the following points. Firstly, the unique clinical feature in this case is lateral knee snapping while pain, swelling, discomfort were reported in previous case reports. During the surgery, we found a cyamella adjacent to myotendinous junction of popliteus tendon and snapping of the popliteus tendon over the cyamella. This is previously unreported in the existing literature. We postulate that the unique clinical feature is associated with the location and size of cyamella. Secondly, we reported surgical excision in dealing with snapping knee caused by symptomatic cyamella for the first time and achieved satisfied outcome after 8 weeks follow-up. The treatment of existing case reports includes physical therapy, hinge brace and arthroscopy, the pathology of snapping is unclear and the clinical outcome is uncertain. Thirdly, we noted that Cabot sign is a typical physical examination for differential diagnosis of the lateral snapping knee, Cabot sign could reproduce the unique snapping while other spacial tests were unremarkable, thus a symptomatic cyamella should be considered as differential diagnosis. Fourthly, we proposed that symptomatic cyamella in the popliteus tendon should be considered as part of the differential diagnosis of the snapping knee. Many differentials for the snapping knee have been reported as mentioned above, symptomatic cyamella in the popliteus tendon causing snapping knee is identified for the first time and should be considered in clinical works.

The reported case is the first that the authors have evaluated with a symptomatic cyamella in the popliteus tendon as an etiology for snapping knee. This was an unexpected finding intraoperatively and was only discovered by directly visualizing and palpating the popliteus tendon snapping over the cyamella during passive range of motion. This case offers a contribution by adding to the differential diagnosis that should be considered in the evaluation of snapping knee syndrome.

Although not previously suggested, symptomatic cyamella in the popliteus tendon should be considered as part of the differential diagnosis of the snapping knee. Although an early diagnosis could be difficult to establish, the typical MRI is an ovoid structure with internal cancellous bone signal in the popliteus tendon. An open excision of the cyamella is the optimum surgical treatment for this condition.

## Supplementary information

**Additional file 1.** A reproducible audible and palpable snapping of the lateral knee when moving from flexion to extension.

**Additional file 2.** In the surgery, we found there was snapping of the popliteus tendon over the cyamella.

**Additional file 3.** After excision surgery, the snapping was gone.

## Data Availability

Not applicable.
